# COVID anomaly in the correlation analysis of S&P 500 market states

**DOI:** 10.1371/journal.pone.0301238

**Published:** 2024-04-18

**Authors:** M. Mijaíl Martínez-Ramos, Manan Vyas, Parisa Majari, Thomas H. Seligman

**Affiliations:** 1 Instituto de Ciencias Físicas - Universidad Nacional Autónoma de México, Cuernavaca, Morelos, México; 2 Centro Internacional de Ciencias AC - UNAM, Avenida Universidad 1001, UAEM, Cuernavaca, Morelos, México; Vilnius University, LITHUANIA

## Abstract

Analyzing market states of the S&P 500 components on a time horizon January 3, 2006 to August 10, 2023, we found the appearance of a new market state not previously seen and we shall discuss its possible implications as an isolated state or as a beginning of a new general market condition. We study this in terms of the Pearson correlation matrix and relative correlation with respect to the S&P 500 index. In both cases the anomaly shows strongly.

## Introduction

Market states, introduced in 2012 [1] on the basis of the correlations of returns, have seen numerous applications in financial market studies and also beyond [2–13]. As far as financial markets are concerned there is some additional information obtained beyond the “State of the Market” associated to the largest eigenvalue of the correlation matrix of returns [14]; this shows some structure in the Market and differences between markets, that might well relate to their efficiency. Also risk assessment in situations associated to crashes are of some interest [5, 7]. Yet these studies indicate that the largest eigenvalue or equivalently the average correlation, which is very strongly correlated with the largest eigenvalue [14, 15], seem to dominate the picture. Dimensionally scaled dynamics have been shown [5–7, 10, 12, 13] and they seem to confirm this idea. The time trajectory in the space of correlation matrices [16] visits the clusters over longer time horizons.

Recently some attention was drawn to the use of projected correlations [10, 12, 13] eliminating the largest eigenvalue which in turn are compared [17] to the use of relative correlations [18, 19]. To some extent this seems to be due to the fact that the number of independent matrix elements is too large to produce a clear picture. A fruitful idea may be to use pattern recognition techniques to visualize these systems. Considering that we have *N* = 322 S&P 500 stocks, in a time horizon January 3, 2006 to August 10, 2023, it would lead to *N*(*N* + 1)/2 = 52, 003 variables. An analysis for market sectors rather than stocks successfully reduces this [20] using coarse grained correlation matrices introduced in [10, 12, 13] to symmetric sectorial matrices. Even these matrices based on ten sectors will produce 55 variables. Further reductions via coarse grainings seem feasible and also show some success [21]. Yet in the last case taken to its extreme of 2 × 2 matrices with but three parameters seems interesting [21] but not entirely satisfying, in particular because of an arbitrariness that arises. In previous work it proved useful to use the power map [22–24] to reduce fluctuations. As we wish to eliminate the effect of the average correlation to emphasize subtler correlations, we shall first proceed without this tool.

In this scenario we found in a new analysis with a time horizon January 3, 2006 to August 10, 2023 that includes the COVID-19 pandemic [25] as well as its aftermath as far as it is known. We shall find a remarkable fact, namely that in 2020 an entirely new state appears that has not appeared in the time interval January 3, 2006 to December 31, 2019. We will call it the “COVID state”. For a period of several months it entirely dominates the picture and then seems to taper off. Among the intriguing features that do appear, is the fact that the COVID state does not appear in March 2020 [25] but about three months later on 1st June 2020 (The exact date depends slightly on the number of states, time horizon and size of the epochs used.). This feature at least can already be well understood, because that period corresponds to a crash, which is well identified with panic sell-off and for which the average correlation is the main factor. This induces us to use the concept of relative correlation [18, 19] and as reference time series, we use the simplest time series, namely the S&P 500 index itself. We also look into the behavior of relative correlations discussed in detail in Ref. [17]. Indeed this relative correlation matrix will now have the COVID state as its dominant component with the largest average relative correlation and with this measure it will start in March 2020. This establishes the appearance of a new market state. What we can not determine is, if the COVID state indicates the beginning of a changed market behavior or if it essentially ends with COVID.

In the next section, we describe the data and techniques we use. The following section gives numerical results for state evolution, transition matrices, distributions of correlation matrix elements over the total time horizon. and participation ratios. Finally, conclusions and an outlook are presented. We also show some illustrations of clustering of correlation matrices in the S1 File [26].

## Data and techniques used

We choose the stocks of S&P 500 index as they represent the most important quoted companies of the US market [27]. From these stocks, we select all those that within the time horizon January 3, 2006 to August 10, 2023 have no more than two consecutive trading days without a quote (*T* = 4431 total trading days). The number of stocks is thus reduced to 322 and the corresponding stocks are listed in the S1 File [26].

For the US-Market, as represented by the stocks making up the S&P 500 index, we find that market states are roughly ordered according to their average correlation as long as we don’t choose too large a time scale for epochs. We divide the total time horizon *T* into epochs of 20 trading days and use logarithmic returns *r* between these days as the dataset, given the adjusted closing price *p*_*i*_(*t*) of trading day *t* for stock *i*,

$$\begin{array}{c} r_{i}\left(t\right)=\text{log}\;[\frac{p_{i}\left(t\right)}{p_{i}\left(t-1\right)}]\;. \end{array}$$ (1)

For the corresponding returns, we assume zero for the days without closing quote while the return for the active trading day is computed using last active trading day. Using these returns time series, we calculate Pearson correlation matrix (For the results presented in the paper, we use the formula for the Pearson correlation matrix elements although, at least for some of the epochs, time series are not even weakly stationary). *C* with matrix elements given by [14, 18]

$$\begin{array}{c} C_{i,j}=\frac{\left\langle r_{i}\;r_{j}\rangle -\langle r_{i}\rangle \langle r_{j}\right\rangle }{\sigma_{i}\;\sigma_{j}}\;, \end{array}$$ (2)

with *σ* is the standard deviation of the respective return time series for the stocks.

The relative correlation *RC* between two return time series *r*_*i*_ and *r*_*j*_ with respect to the S&P 500 index returns time series *r*_*SP*_ is defined as,

$$\begin{array}{c} RC_{ij:SP}=\frac{C_{i,j}-C_{i,SP}\;C_{j,SP}}{{\left[(1-C_{i,SP}^{2})(1-C_{j,SP}^{2})\right]}^{1/2}}\;. \end{array}$$ (3)

Here, *C*_*i*,*j*_ are the Pearson correlation coefficients defined by Eq (2).

Using the S&P 500 data from January 3rd 2006 to August 10th 2023, we divide the total time horizon (*T* = 4431 total trading days) in epochs of 20 trading days with one day shift and we shall analyze the time evolution of market states by clustering 322 × 322 dimensional correlation matrices of the 322 stocks that were quoted throughout the time horizon with interruption no longer than two consecutive trading days. For the corresponding returns, we assume zero for the days without closing quote, while the return for the active trading day is computed using last active trading day. We use the *k*-means clustering formalism following the lines of [5, 17] and show the case of five and six market states which seem appropriate for the S&P 500 data; other state numbers will be discussed in the S1 File [26]. We have verified the robustness of COVID state using the *k*-means clustering by increasing the number of clusters from 5 to 12, both for the Pearson and relative correlations. Also, we assign the average correlation of the cluster as the specific property of the state.

## Results and discussion

### Time evolution of market states

**Fig 1:**
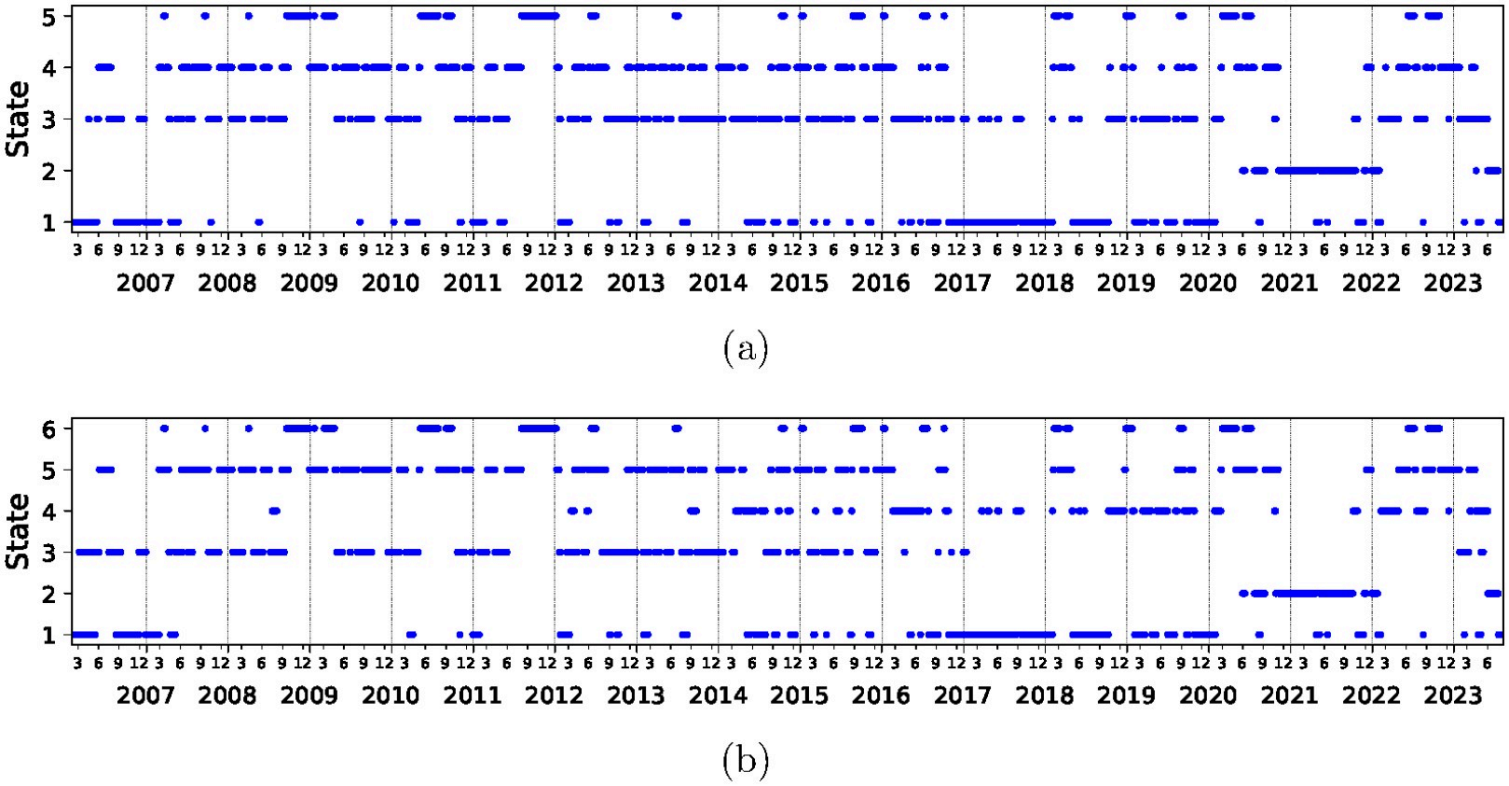
Time evolution of market states of the S&P 500 data using Pearson correlation matrix *C* defined by Eq (2) in a time horizon from January 3rd 2006 to August 10th 2023 with an epoch of 20 trading days. Pearson correlation matrix elements are computed using logarithmic return time series of adjusted closing prices. Frame (a) and (b) show the cases of five and six states, respectively. The market states are arranged in order of increasing average correlations. The average correlations for the states are (a) 0.17, 0.27, 0.30, 0.44, 0.61 and (b) 0.16, 0.26, 0.28, 0.31, 0.44, 0.61, respectively.

The principal result of this paper is seen in Fig 1 where the time sequence of the five and six states is displayed. Indeed, these figures show one very notable feature. State 2 does not appear before June 1st 2020 and then almost uninterruptedly dominates the situation until February 1st 2022 where it peters out. As the clusters are numbered according to the average correlation it is clear that state 2 corresponds to fairly low average correlation of ∼0.26 but is separated from other low average correlation states. For comparison, we also show the market evolution for time period between January 3rd 2006 to December 31st 2019 in S1 File [26]. Also shown in S1 File [26] is the 3D view of the correlation matrices after subjecting them to dimensional scaling according to the recipe given in [28].

**Fig 2:**
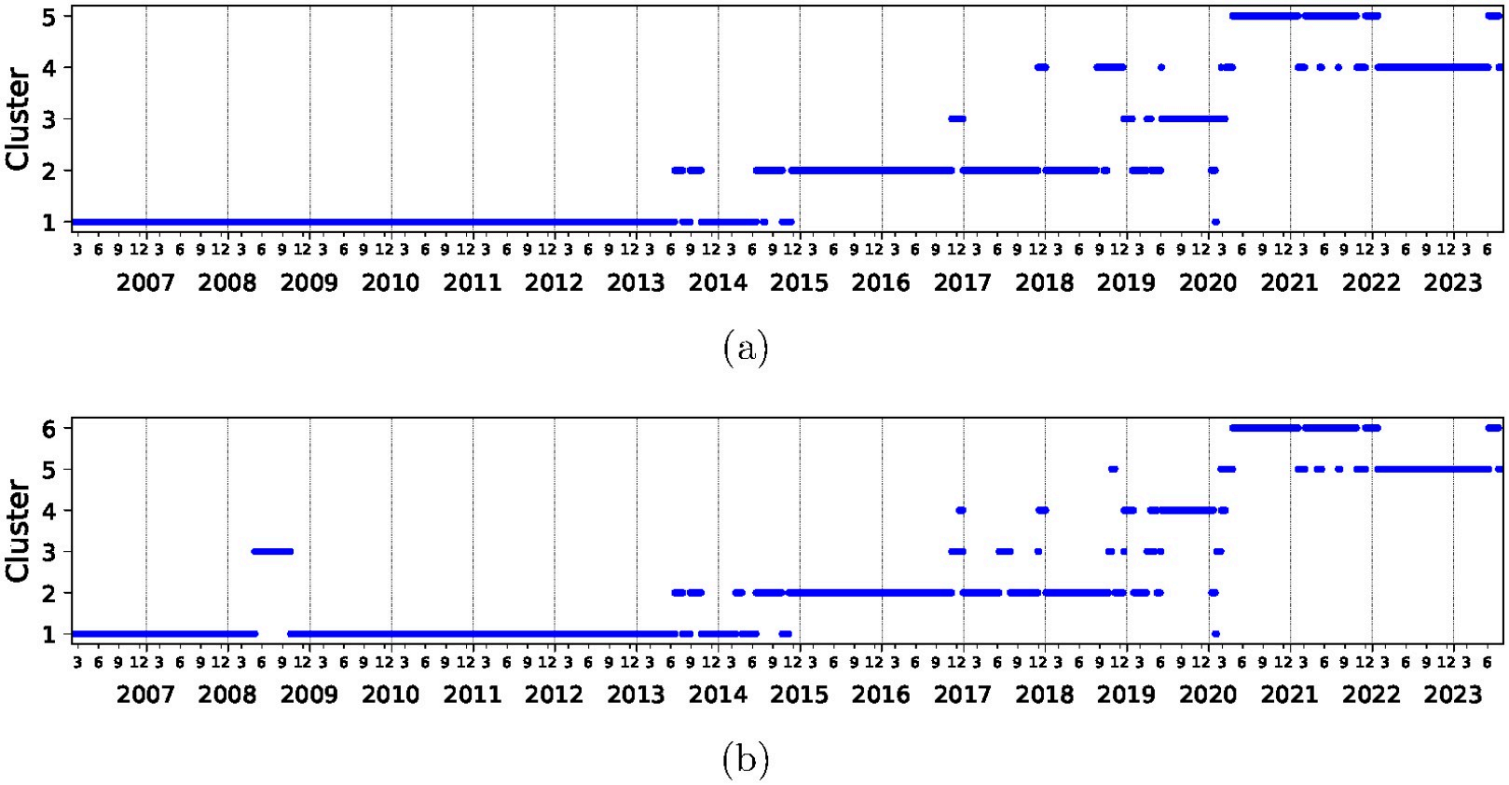
Clustering image of the evolution of the relative correlations *RC* with respect to S&P 500 index defined by Eq (3) with time horizon and epoch length as in Fig 1. The clusters are arranged in order of increasing average relative correlations. The average relative correlations for the clusters are (a) 0.014, 0.015, 0.019, 0.042, 0.083 and (b) 0.013, 0.015, 0.018, 0.024, 0.048, 0.084, respectively. Note that the cluster 5 in frame (a) and cluster (6) in frame (b) start approximately three months earlier than the start date of state 2 in Fig 1.

COVID started in March 2020 but the corresponding state 2 of Pearson correlation *C*, defined in Eq (2), appears only in June 2020. There is a simple explanation for this as the initial financial panic of COVID ended in June 2020. This behavior is associated to the largest eigenvalue and we expect the S&P 500 index to reflect that. We therefore look at the evolution of market using relative correlations *RC*, defined in Eq (3), and show the results in Fig 2. We see that the COVID state is rather isolated but now begins in March 2020. Otherwise, the properties of this state are rather similar—this state will begin in March 2020 and peter out at the approximately same time as with *C*. The main difference between the corresponding states is the different starting date. Indeed this state shows the highest average relative correlation and therefore, may also be an important tool to find additional relevant variables for the market, besides the highest eigenvalue of the Pearson correlation matrix. Other techniques to identify the subtler correlations are discussed in detail in [17].

### Transition matrices

**Fig 3:**
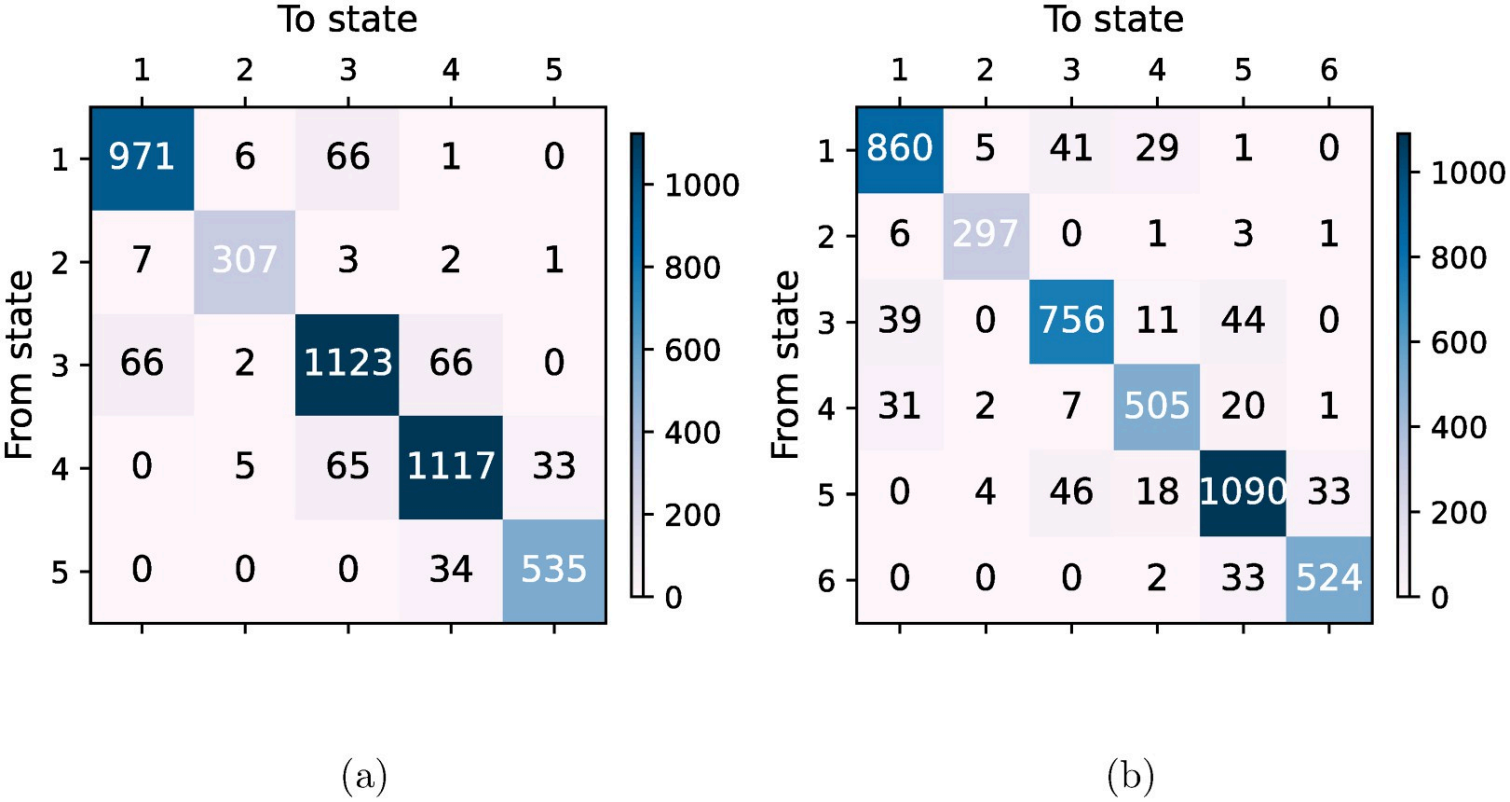
Transition matrices showing jumps between different market states shown in Fig 1 with (a) five clusters and (b) six clusters. The transition matrices are nearly tri-diagonal and show the state 2 distinctively. The necessary Markovianity criterion given in Eq. (2) of [5] is fulfilled. The equilibrium distributions corresponding to (a) and (b) are (0.237, 0.073, 0.285, 0.277, 0.129) and (0.212, 0.069, 0.193, 0.128, 0.270, 0.127) respectively.

At this point we could go two ways. Either explore further properties of the states and their transitions or try an economic explanation. The latter is at the margin of our knowledge and thus we further explore the unusual dynamics we encounter. Next step is to look at the transition matrices, as shown in Fig 3. The transition matrices are nearly tri-diagonal and show the COVID state distinctively. The necessary Markovianity criterion given in Eq. (2) of [5] is fulfilled. The equilibrium distributions corresponding to Fig 3(a) and 3(b) are (0.237, 0.073, 0.285, 0.277, 0.129) and (0.212, 0.069, 0.193, 0.128, 0.270, 0.127) respectively. Note that state 2 has few transitions as can be seen from Fig 1, a signature we have never found before. This reinforces the interest in the COVID state. The transitions are principally located at the edges of state 2, which indicates that it is essentially a smooth transition. It is important to mention that for risk assessment, noise suppression techniques applied to the correlation matrix rather than to time series [22–24] are important.

### Distribution of correlation matrix elements over total time horizon

We note that this anomaly appears during the main COVID period and we may suspect that it is hidden by the panic at the beginning of this pandemia which implies high correlations as we can again see from Fig 1. Therefore, we will look at the relative correlations *RC* with respect to the S&P 500 index as defined in Eq (2) to explore if these display features of state 2 also during the panic period at the beginning or the slump of S&P 500 at the beginning of the pandemic. We inspected results [17] obtained in this context previously and distribution of the correlation matrix elements for each epoch turns out to be of particular interest.

**Fig 4:**
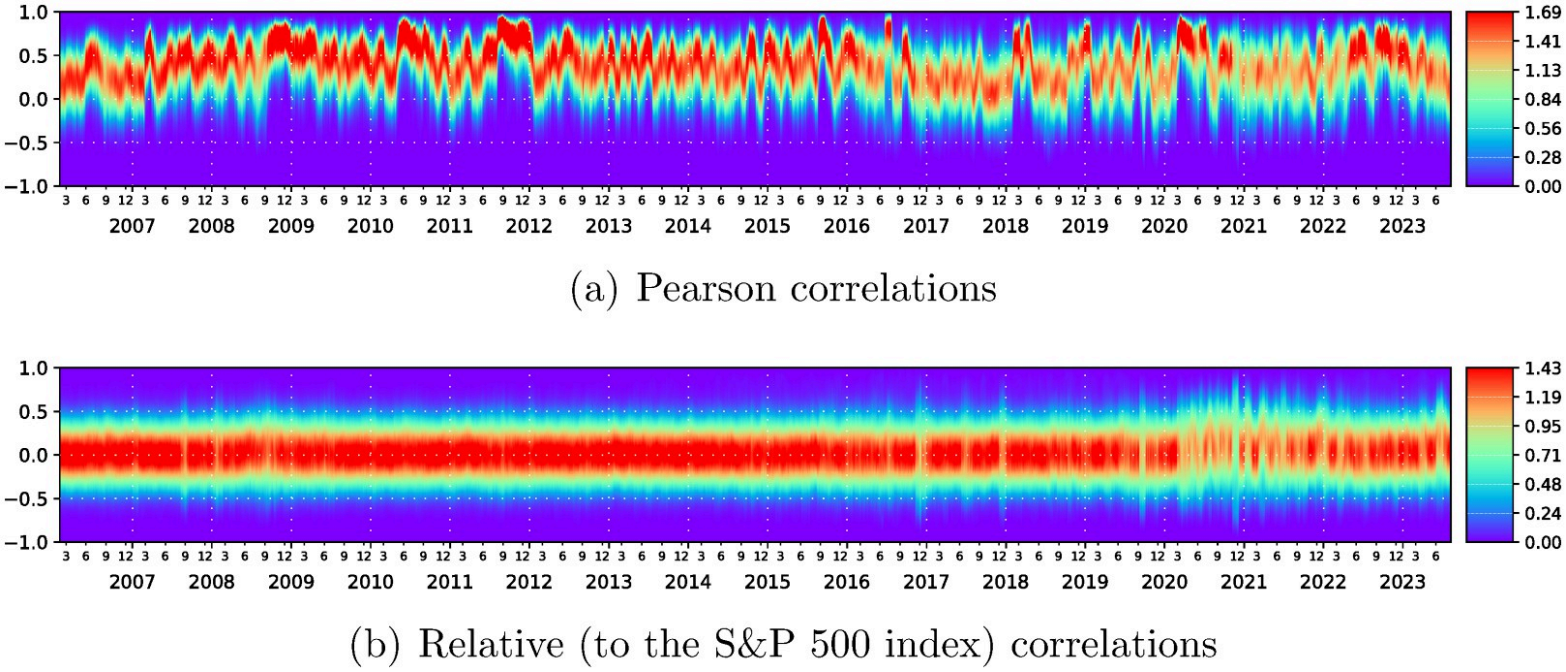
Time evolution of distribution of correlation matrix elements corresponding to (a) Pearson correlation coefficients defined by Eq (2) and (b) relative (with respect to the S&P 500 index) correlation coefficients defined by Eq (3). For the time period starting June 1st 2020 where state number 2 starts, the fluctuation of the matrix elements of *C* becomes much faster and does not stop at the end of state 2 (February 1st 2022) but persists. Also, this behavior does not start at the beginning of COVID period but at the beginning of state 2. Whereas with *RC*, the state starts in March 2020.

We found striking results when looking at the histograms for distribution of correlation matrix elements for each of the epochs as shown in Fig 4. A more detailed analysis will be given in [17] but a simple ocular inspection shows two points: For the time period starting June 1st 2020 where state number 2 starts, the fluctuation of the matrix elements become much faster and this first sight behavior does not stop at the end of state 2 but persists. This behavior does not start at the beginning of COVID period but at the beginning of state 2. Indeed it starts when the crash of the stock market and strong following fluctuations approximately end due to panic that can be seen between November 9th 2020 and February 1st 2022 can override the COVID influence to some extent. We therefore relate this to data we obtained in an almost concluded analysis [17] of relative [18, 19] and reduced [10, 12, 13] correlation matrices. We see markedly different behavior that with beginning of COVID, we have a change in the behavior of market as long as the highest correlation does not dictate it.

### Participation ratios

Participation ratios (PR) gives the number of components that participate significantly in each eigenvector *v*,

$$\begin{array}{c} PR_{v}=[\sum_{i=1}^{N}{\left|v_{i}\right|}^{4}]\;. \end{array}$$ (4)

**Fig 5:**
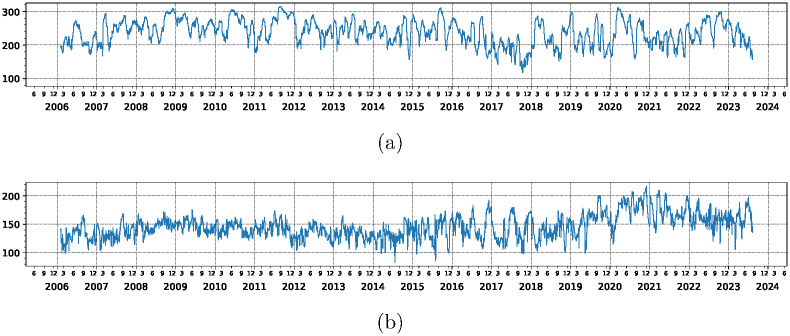
Time evolution of PR defined in Eq (4) for eigenvector of the largest eigenvalue corresponding to (a) Pearson correlation coefficients defined by Eq (2) and (b) relative (with respect to the S&P 500 index) correlation coefficients defined by Eq (3). The vertical shadowed stripes indicate market crash periods which are usually mentioned in the literature.

PR takes values between 1 and *N* and for a Gaussian Orthogonal Ensemble (GOE) has the limiting value of *N*/3 [29–31]. This GOE result holds true for correlation matrices as well and will be seen in the center of the spectrum for sufficiently long epochs. We analyze the time evolution of PR corresponding to the largest eigenvalues using Pearson and relative (with respect to the S&P 500 index) correlation coefficients respectively in Fig 5(a) and 5(b). For the Pearson correlations, the PR is above the GOE threshold (107.33) for all the epochs. However, for the relative correlations, in the COVID period, the PR is constantly high in comparison to other epochs.

**Fig 6:**
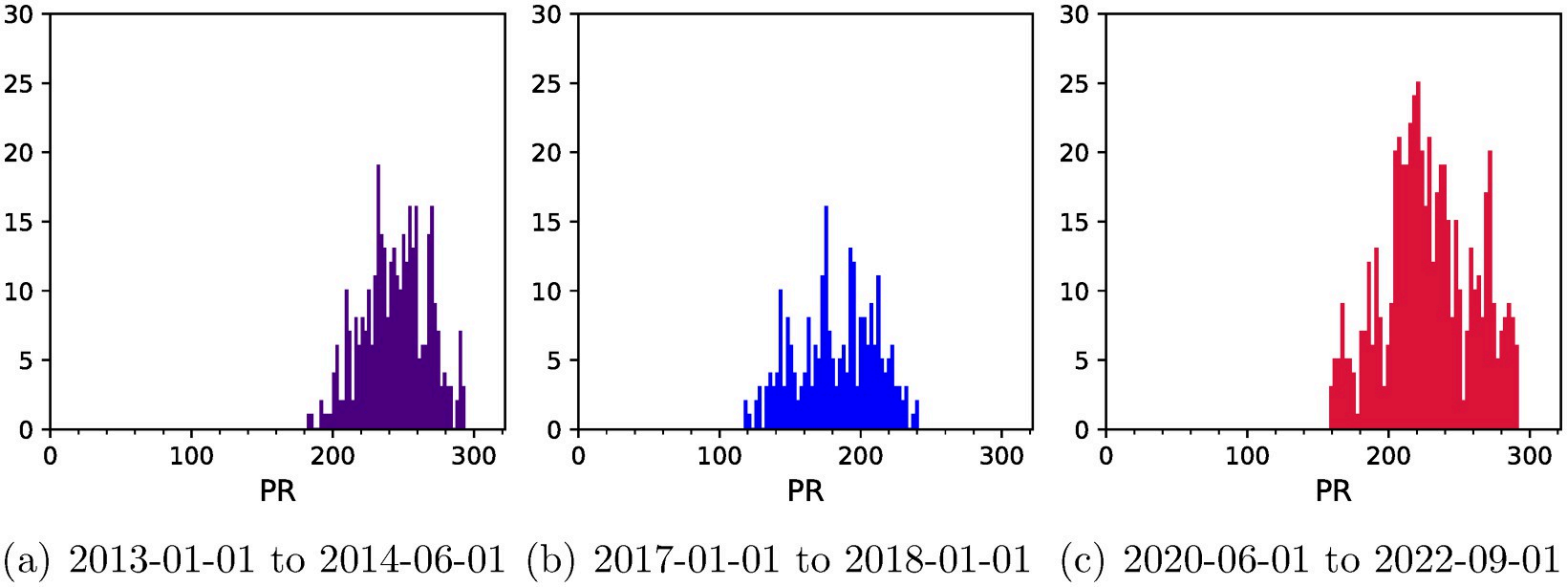
Distribution of PR defined in Eq (4) for eigenvector of the largest eigenvalue corresponding to Pearson correlation coefficients defined by Eq (2) for three different time periods—(a) non-calm period, (b) calm period, and (c) COVID period. Note that the format for the dates used is YYYY-MM-DD. The first four moments (average, variance, skewness, kurtosis) are as follows—(a) (244.5, 525.04, -0.13, -0.48), (b) (182.34, 799.19, -0.17, -0.83), and (c) (228.66, 1008.69, -0.004, -0.65). Note that these figures have same scales.

To probe into further details, we choose three different time periods within our time horizon: (a) 2013–01-01 to 2014–06-01, (b) 2017–01-01 to 2018–01-01, and (c) 2020–06-01 to 2022–09-01, corresponding respectively to non-calm period, calm period and the COVID period. We then analyze the histograms for PR for these three time periods as shown in Fig 6, obtained using Pearson correlation matrices. The average of the distribution is highest for the non-calm period, lowest for the calm period, and intermediate for the COVID period. However, the variance and skewness are largest for the COVID state. The distribution of PR in the COVID period is quite symmetrical, unlike the other two time periods chosen. We also looked at Inverse Participation Ratios (IPR) and the signal is less clear. This is not surprising as the IPR is used for analytical purposes as these are entire functions and PR is a natural choice for data analysis. The statistical analysis of eigenvalues is not conclusive due to reduced sample sizes in the shorter time periods.

## Conclusions and future outlook

Starting from a multivariate correlation analysis of financial markets using a methodology that resulted in the definition of market states [1] and using the specific techniques proposed in [5, 7], we found that a previously non-existent market state appears in a time frame closely related to the COVID pandemic. Expanding the methodology by using also the relative correlations of stocks with respect to the market index, we get results that we hope will give a deeper insight into the concept of market states. The emergence of the “state of the market” represented by the largest eigenvalues for both Pearson and relative correlations seems noteworthy. The stability of the results is confirmed by the corresponding discussions in the S1 File [26]. For four states, the COVID state is not visible. Going beyond four, we have shown results for 5–8 states that the qualitative behavior of COVID state remains unchanged. Although we would like to remark that it might split up increasing the states further. We can not use an arbitrary large number of states but we checked up to 12 states that the COVID state is qualitatively unchanged. Relative correlations show similar behavior. The temporal coincidence makes us believe that it has to do with economical consequences of the restrictions and the mindset of the population during the COVID pandemic. This idea is fortified by the observation that the full correlation matrix analysis indicates an onset of the COVID state roughly three months after the onset of this state, at which time panic sales and the corresponding crash associated with high average correlation are over. This state ends in February 2022 with a few points reappearing at the end of our time horizon. Being at the end of our time horizon these points are not very reliable, but at any rate we cannot yet distinguish if we are talking of a very specific and time bound reaction to COVID or whether we see a new general market situation. Time might tell.

It is remarkable that the COVID state is clearly marked as the state with the highest average correlation relative to the S&P 500 index. Note though that the beginning of this state for the relative correlations is roughly coinciding with the strong crash of the stock values while this state for the Pearson correlations appears towards the end of COVID crash. This indicates that the high correlation still dominates the market but once the average correlation decreases, other components become important. These other components are visible if the general market behavior is removed using relative to the S&P 500 index correlations. This increases the relevance of the very concept of relative correlation in financial markets and indeed relates also to recently developed concepts of reduced correlations by Guhr and co-workers [10, 12, 13]. We present more details about these methods and results in Ref. [17]. Indeed we hope that this example will help us in our search for relevant parameters in the stock market beyond the highest eigenvalue of the Pearson correlation matrix (essentially equivalent to the average correlation) yet significantly smaller in number than the huge number of matrix elements of the correlation matrix [20, 21]. We do not use power map [22–24] for noise suppression as we want to emphasize subtler correlations. This may even lead to the use of an “anti Power map” i.e. with powers smaller than one.

## Supporting information

S1 File (PDF)

S1 Video (MP4)

S2 Video (MP4)

S3 Video (MP4)
