# Supplementary material for: COVID anomaly in the correlation analysis of S&P 500 market states
Source: PLoS One. 2024 Apr 18;19(4):e0301238. doi: 10.1371/journal.pone.0301238 (PMC11025781; doi:10.1371/journal.pone.0301238)
Supplement: S1 File — (PDF) [file pone.0301238.s001.pdf]

Supplemental Material: COVID anomaly in the correlation analysis of S&P 500 market states

M. Mijaíl Martínez-Ramos[1], Manan Vyas[1*], Parisa Majari[1], Thomas H. Seligman[1, 2]

1 Instituto de Ciencias Físicas - Universidad Nacional Autónoma de México, Cuernavaca, 62210, Morelos, México
2 Centro Internacional de Ciencias AC - UNAM, Avenida Universidad 1001, UAEM, Cuernavaca, 62210, Morelos, México

* manan@icf.unam.mx (MV)

Classification of the S&P 500 market states into (a) five and (b) six clusters displays the atypical state 2 corresponding to COVID anomaly. To further substantiate the existence of this state, we compare the time evolution of market states starting January 3rd 2006 to (a) December 31st 2019 and (b) August 10th 2023 in Figures S1(a) and S1(b) respectively. Also, the structure of the transition matrices display this signature clearly as shown in Figs. S2(a) and S2(b). The necessary Markovianity criterion given in Eq. (2) of [1] is also fulfilled by the transition matrices. The equilibrium distributions are $(0.240, 0.073, 0.285, 0.277, 0.129)$ and $(0.212, 0.069, 0.193, 0.128, 0.270, 0.127)$ respectively for five and six market states.

This immediately makes us suspect that the linear alignment seen in the dimensionally scaled picture of all the correlation matrices is no longer conserved [1, 2]. We therefore show in Figures S3, S4, and S5 the Pearson correlation matrices with 5 market states of the S&P 500 data from January 3rd 2006 to December 31st 2019, 5 market states of the S&P 500 data from January 3rd 2006 to August 10th 2023, 6 market states of the S&P 500 data from January 3rd 2006 to August 10th 2023, respectively, after subjecting them to dimensional scaling according to the recipe given in [3]. These figures show a single frame from the video, click in the captions to play the videos. We scale down to 3 dimensions and we see that the usually rather smooth picture shows a bulge in Figure S4 which under close scrutiny results to correspond exactly to state 2 as demonstrated by the color code of the picture.

We also analyze the existence of state corresponding to COVID anomaly by increasing the number of clusters and the results are as shown in Fig. S6. Although we show results only for seven and eight clusters, we have verified that this state exists for $9, 10, 11$ and 12 clusters as well. This illustrates that the atypical state corresponding to COVID is stable.

Fig S1. Time evolution of market states of the S&P 500 data using Pearson correlation coefficients in a time horizon from January 3rd 2006 to (a) December 31st 2019 and (b) August 10th 2023, with an epoch of 20 trading days. Pearson correlation matrix elements are computed using logarithmic return time series of adjusted closing prices. The market states are arranged in order of increasing average correlations. The average correlations for the market states are (a) 0.16, 0.28, 0.30, 0.43, 0.60 and (b) 0.17, 0.26, 0.30, 0.44, 0.61, respectively. State 2 corresponding to COVID anomaly shows up in the time period starting June 1, 2020 until February 1, 2022.

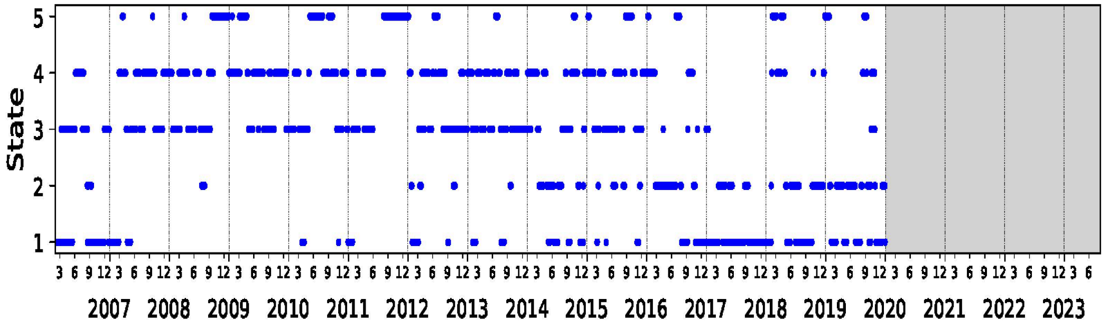

(a)

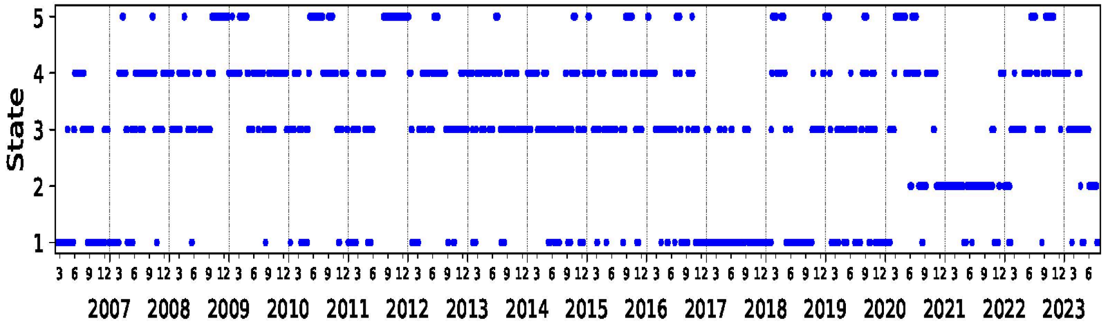

(b)

Fig S2. Transition matrices corresponding to Pearson correlation coefficients showing transitions between different market states of the S&P 500 data from January 3rd 2006 to (a) December 31st 2019 and (b) August 10th 2023. The difference due to appearance of COVID anomaly is visible in transition matrix as well. Also, the necessary Markovianity criterion given in Eq. (2) of [1] is fulfilled. The equilibrium distributions corresponding to time periods of (a) and (b) are $(0.228, 0.224, 0.131, 0.286, 0.1308)$ and $(0.240, 0.073, 0.285, 0.277, 0.129)$ respectively.

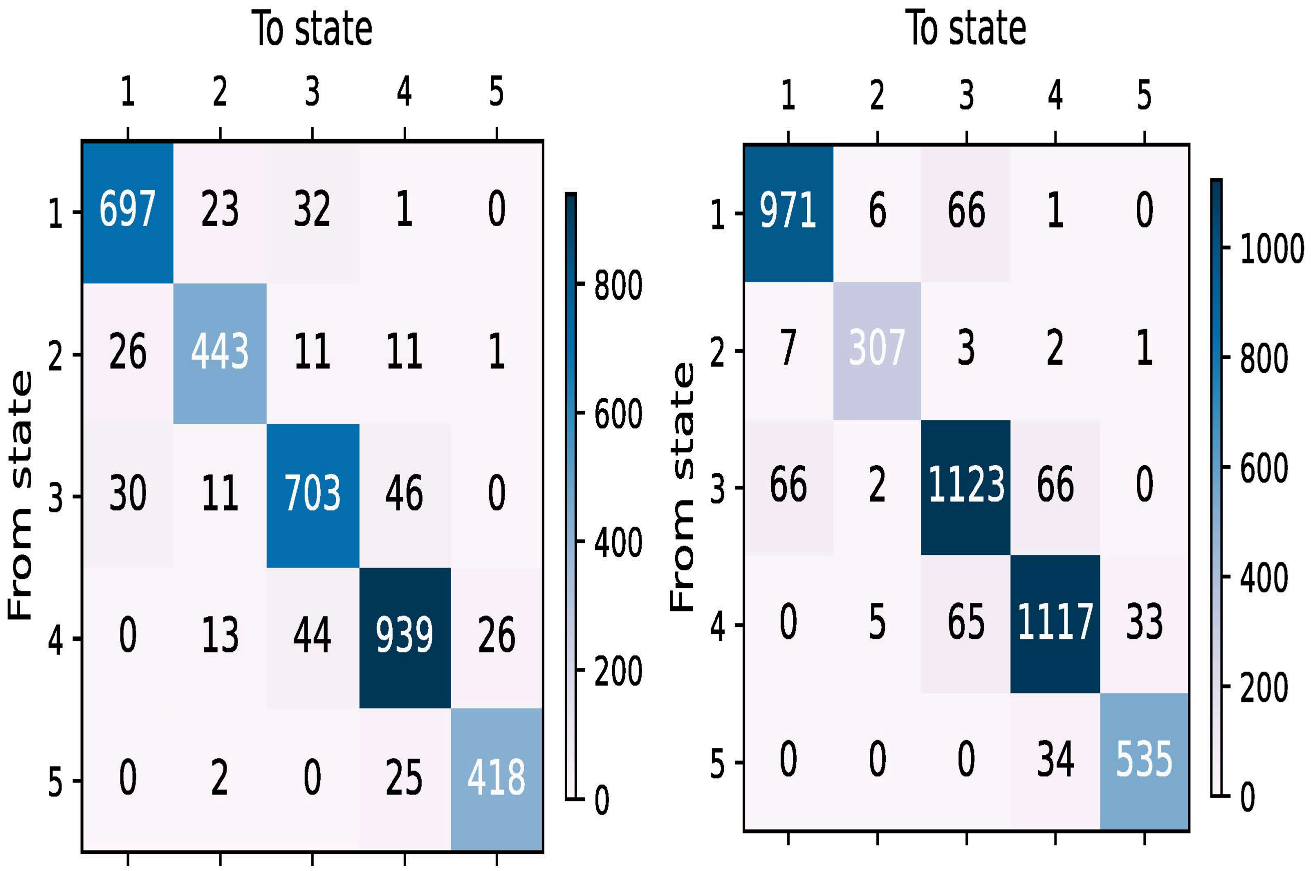

(a) (b)

Fig S3. L^1 3D Multi Dimensional Scaling after k-means clustering of 3503 Pearson correlation matrices (projections on principal components) of the five S&P 500 market states from January 3rd 2006 to December 31st 2019. The image above shows a single frame from the video. Click Vid1 to play the video.

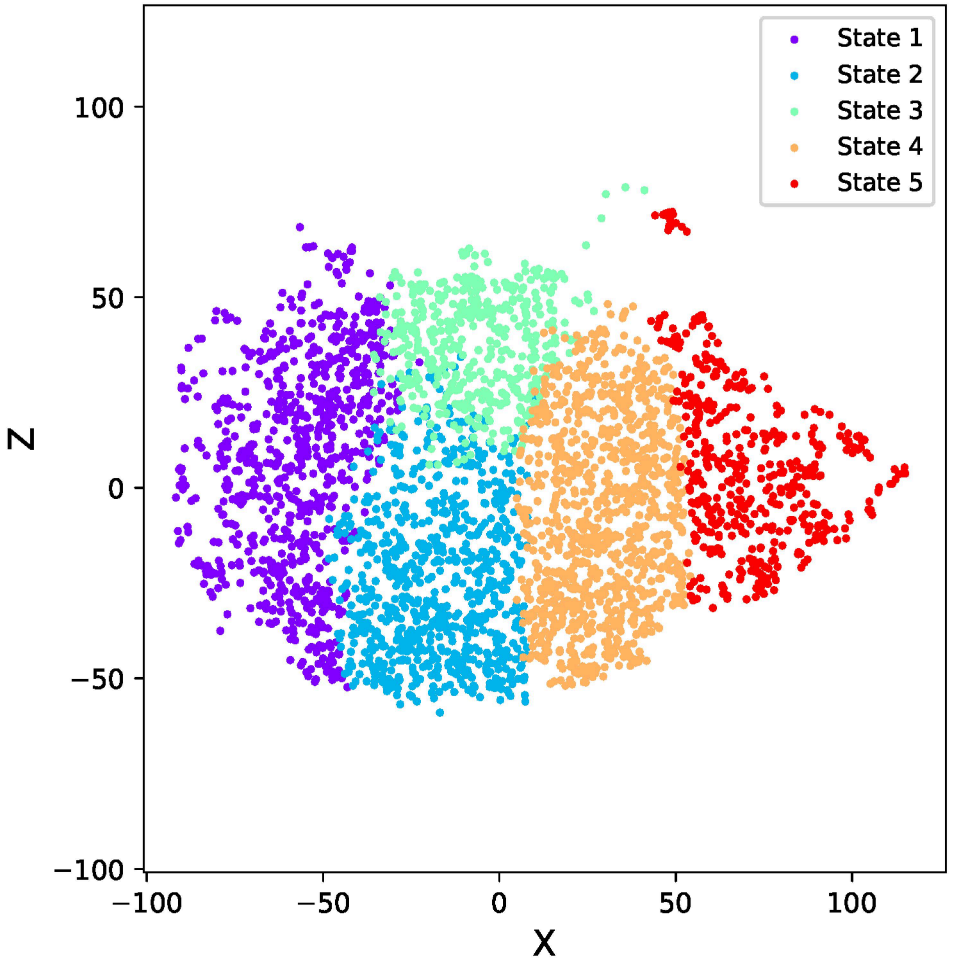

Fig S4. L^1 3D Multi Dimensional Scaling after k-means clustering of 4411 Pearson correlation matrices (projections on principal components) of the five S&P 500 market states from January 3rd 2006 to August 10th 2023. The image above shows a single frame from the video. Click Vid2 to play the video.

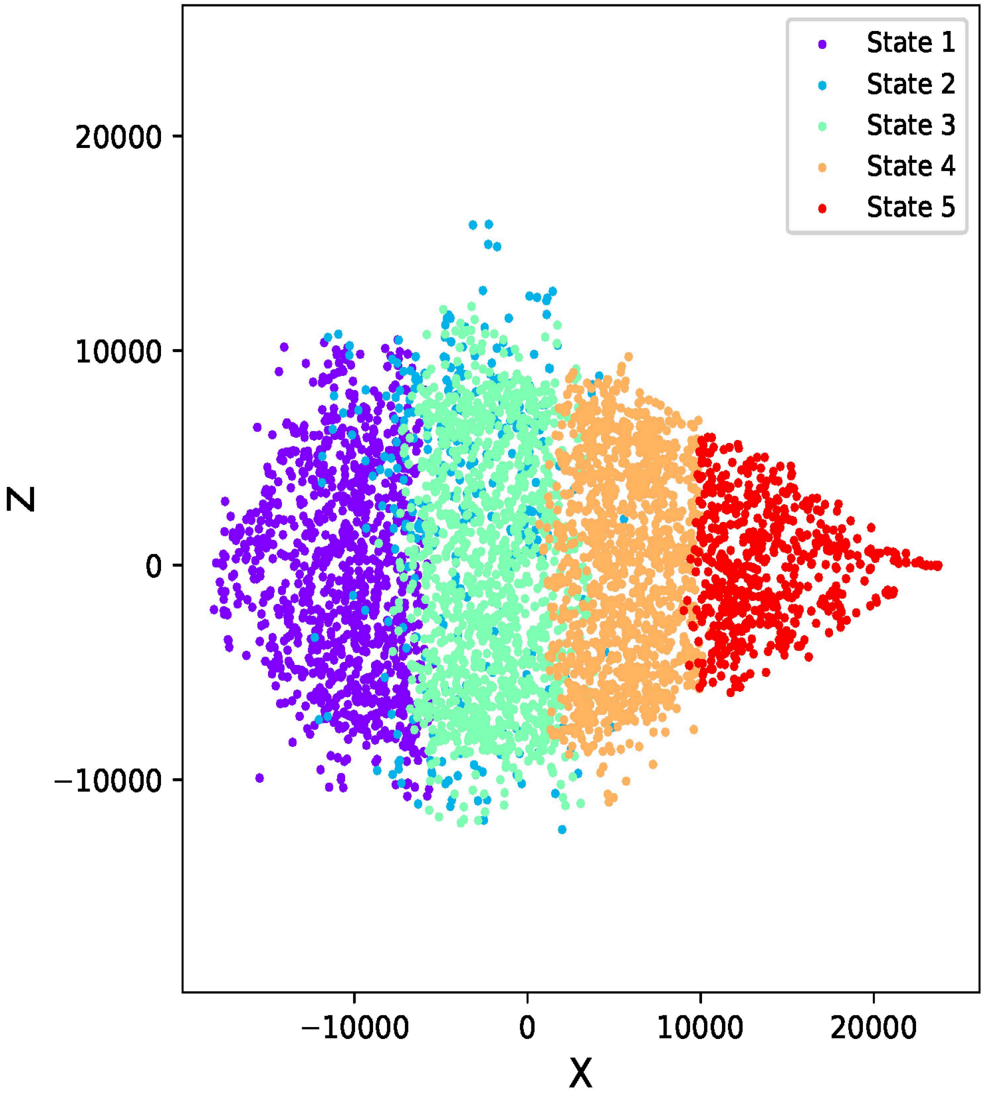

Fig S5. L^1 3D Multi Dimensional Scaling after k-means clustering of 4411 Pearson correlation states (projections on principal components) of the six S&P 500 market states from January 3rd 2006 to August 10th 2023. The image above shows a single frame from the video. Click Vid3 to play the video.

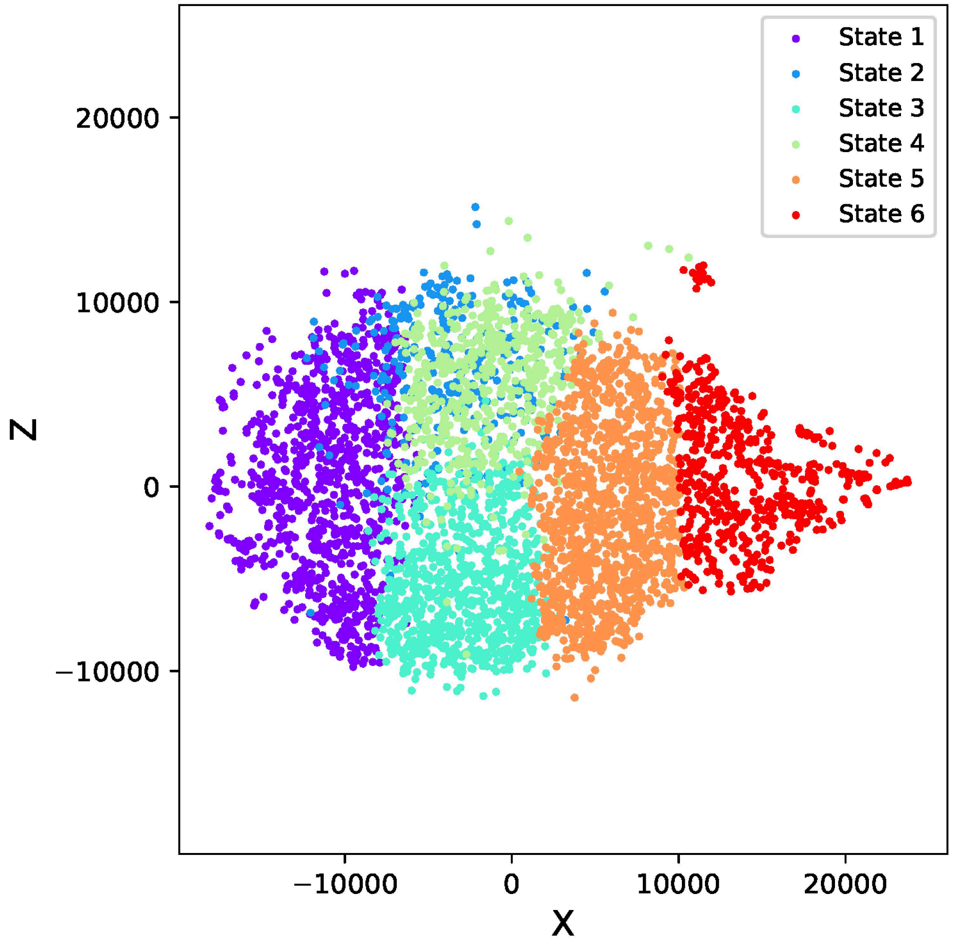

Fig S6. Time evolution of market states of the S&P 500 data using Pearson correlation coefficients in a time horizon from January 3rd 2006 to August 10th 2023 for (a) seven and (b) eight clusters. Pearson correlation matrix elements are computed using logarithmic return time series of adjusted closing prices. The market states are arranged in order of increasing average correlations. The average correlations for the market states are (a) 0.15, 0.24, 0.26, 0.30, 0.39, 0.49, 0.65 and (b) 0.15, 0.20, 0.26, 0.30, 0.31, 0.42, 0.52, 0.66, respectively.

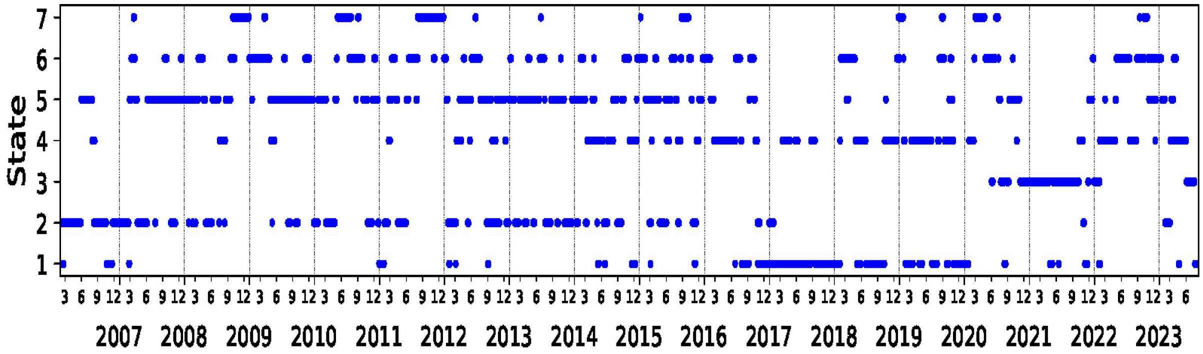

(a) 7 clusters

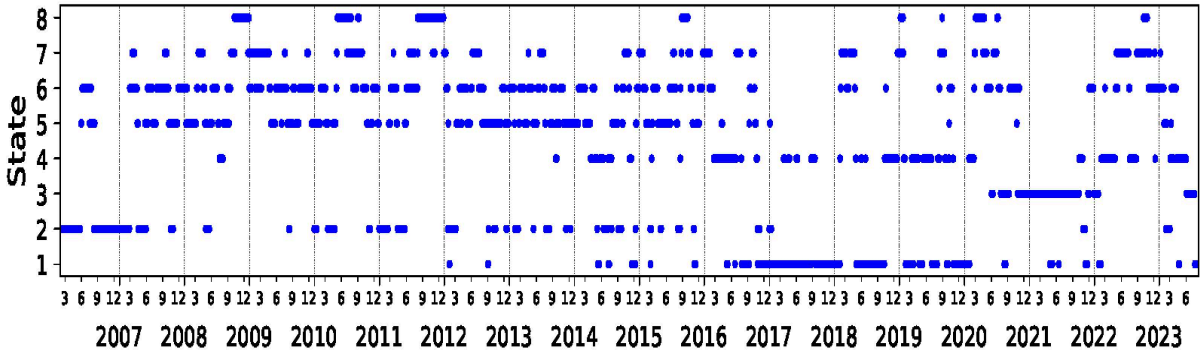

(b) 8 clusters

References

1. H. K. Pharasi, K. Sharma, R. Chatterjee, A. Chakraborti, F. Leyvraz, T. H. Seligman, Identifying long-term precursors of financial market crashes using correlation patterns, New J. Phys. 20 (10) (2018) 103041. doi:10.1088/1367-2630/aae7e0.

2. H. K. Pharasi, S. Sadhukhan, P. Majari, A. Chakraborti, T. H. Seligman, Market state dynamics in correlation matrix space, in: Quantum decision theory and complexity modelling in economics and public policy, Springer, in press 2023.

3. I. Borg, P. Groenen, Modern Multidimensional Scaling: Theory and Applications, Springer Series in Statistics, Springer New York, 2005. URL https://books.google.com.mx/books?id=duTODldZzRcC

List of the 322 stocks analyzed

Sector	Ticker	Name
Basic Materials	APD	Air Products and Chemicals, Inc.
Basic Materials	CF	CF Industries Holdings, Inc.
Basic Materials	ECL	Ecolab Inc.
Basic Materials	FCX	Freeport-McMoRan Inc.
Basic Materials	FMC	FMC Corporation
Basic Materials	IFF	International Flavors & Fragrances Inc.
Basic Materials	MOS	The Mosaic Company
Basic Materials	NEM	Newmont Corporation
Basic Materials	NUE	Nucor Corporation
Basic Materials	PPG	PPG Industries, Inc.
Basic Materials	SHW	The Sherwin-Williams Company
Basic Materials	VMC	Vulcan Materials Company
Communication Services	ATVI	Activision Blizzard, Inc.
Communication Services	CMCSA	Comcast Corporation
Communication Services	DISH	DISH Network Corporation
Communication Services	EA	Electronic Arts Inc.
Communication Services	GOOG	Alphabet Inc.
Communication Services	GOOGL	Alphabet Inc.
Communication Services	IPG	The Interpublic Group of Companies, Inc.
Communication Services	NFLX	Netflix, Inc.
Communication Services	OMC	Omnicom Group Inc.
Communication Services	T	AT&T Inc.
Communication Services	TTWO	Take-Two Interactive Software, Inc.
Communication Services	VZ	Verizon Communications Inc.
Consumer Cyclical	AAP	Advance Auto Parts, Inc.
Consumer Cyclical	AMZN	Amazon.com, Inc.
Consumer Cyclical	AVY	Avery Dennison Corporation
Consumer Cyclical	AZO	AutoZone, Inc.
Consumer Cyclical	BBY	Best Buy Co., Inc.
Consumer Cyclical	BKNG	Booking Holdings Inc.
Consumer Cyclical	CCL	Carnival Corporation & plc
Consumer Cyclical	DHI	D.R. Horton, Inc.
Consumer Cyclical	EBAY	eBay Inc.
Consumer Cyclical	EXPE	Expedia Group, Inc.
Consumer Cyclical	F	Ford Motor Company
Consumer Cyclical	GPC	Genuine Parts Company
Consumer Cyclical	GPS	The Gap, Inc.
Consumer Cyclical	HAS	Hasbro, Inc.
Consumer Cyclical	HD	The Home Depot, Inc.
Consumer Cyclical	HOG	Harley-Davidson, Inc.
Consumer Cyclical	HRB	H&R Block, Inc.
Consumer Cyclical	IP	International Paper Company
Consumer Cyclical	JWN	Nordstrom, Inc.
Consumer Cyclical	KMX	CarMax, Inc.
Consumer Cyclical	KSS	Kohl's Corporation
Consumer Cyclical	LEG	Leggett & Platt, Incorporated
Consumer Cyclical	LEN	Lennar Corporation
Consumer Cyclical	LKQ	LKQ Corporation
Consumer Cyclical	LOW	Lowe's Companies, Inc.
Consumer Cyclical	M	Macy's, Inc.
Consumer Cyclical	MAR	Marriott International, Inc.
Consumer Cyclical	MCD	McDonald's Corporation
Consumer Cyclical	MGM	MGM Resorts International
Consumer Cyclical	MHK	Mohawk Industries, Inc.
Consumer Cyclical	NKE	NIKE, Inc.
Consumer Cyclical	ORLY	O'Reilly Automotive, Inc.
Consumer Cyclical	PHM	PulteGroup, Inc.
Consumer Cyclical	PKG	Packaging Corporation of America
Consumer Cyclical	PVH	PVH Corp.
Consumer Cyclical	RL	Ralph Lauren Corporation
Consumer Cyclical	ROST	Ross Stores, Inc.
Consumer Cyclical	SBUX	Starbucks Corporation
Consumer Cyclical	SEE	Sealed Air Corporation
Consumer Cyclical	TJX	The TJX Companies, Inc.
Consumer Cyclical	TPR	Tapestry, Inc.
Consumer Cyclical	UAA	Under Armour, Inc.
Consumer Cyclical	VFC	V.F. Corporation
Consumer Cyclical	WHR	Whirlpool Corporation
Consumer Cyclical	WYNN	Wynn Resorts, Limited
Consumer Cyclical	YUM	Yum! Brands, Inc.
Consumer Defensive	ADM	Archer-Daniels-Midland Company
Consumer Defensive	CAG	Conagra Brands, Inc.
Consumer Defensive	CHD	Church & Dwight Co., Inc.
Consumer Defensive	CL	Colgate-Palmolive Company
Consumer Defensive	CLX	The Clorox Company
Consumer Defensive	COST	Costco Wholesale Corporation
Consumer Defensive	CPB	Campbell Soup Company
Consumer Defensive	DLTR	Dollar Tree, Inc.
Consumer Defensive	EL	The Estée Lauder Companies Inc.
Consumer Defensive	GIS	General Mills, Inc.
Consumer Defensive	HRL	Hormel Foods Corporation
Consumer Defensive	HSY	The Hershey Company
Consumer Defensive	K	Kellogg Company
Consumer Defensive	KMB	Kimberly-Clark Corporation
Consumer Defensive	KO	The Coca-Cola Company
Consumer Defensive	KR	The Kroger Co.
Consumer Defensive	MDLZ	Mondelez International, Inc.
Consumer Defensive	MKC	McCormick & Company, Incorporated
Consumer Defensive	MNST	Monster Beverage Corporation
Consumer Defensive	MO	Altria Group, Inc.
Consumer Defensive	NWL	Newell Brands Inc.
Consumer Defensive	PEP	PepsiCo, Inc.
Consumer Defensive	PG	The Procter & Gamble Company
Consumer Defensive	SJM	The J. M. Smucker Company
Consumer Defensive	STZ	Constellation Brands, Inc.
Consumer Defensive	SYY	Sysco Corporation
Consumer Defensive	TAP	Molson Coors Beverage Company
Consumer Defensive	TGT	Target Corporation
Consumer Defensive	TSN	Tyson Foods, Inc.

Consumer Defensive	WMT	Walmart Inc.
Energy	APA	APA Corporation
Energy	COP	ConocoPhillips
Energy	CVX	Chevron Corporation
Energy	DVN	Devon Energy Corporation
Energy	EOG	EOG Resources, Inc.
Energy	FTI	TechnipFMC plc
Energy	HAL	Halliburton Company
Energy	HES	Hess Corporation
Energy	HP	Helmerich & Payne, Inc.
Energy	MRO	Marathon Oil Corporation
Energy	NOV	NOV Inc.
Energy	OKE	ONEOK, Inc.
Energy	PXD	Pioneer Natural Resources Company
Energy	SLB	Schlumberger Limited
Energy	VLO	Valero Energy Corporation
Energy	WMB	The Williams Companies, Inc.
Energy	XOM	Exxon Mobil Corporation
Financial Services	AFL	Aflac Incorporated
Financial Services	AIG	American International Group, Inc.
Financial Services	AIZ	Assurant, Inc.
Financial Services	AJG	Arthur J. Gallagher & Co.
Financial Services	AMG	Affiliated Managers Group, Inc.
Financial Services	AMP	Ameriprise Financial, Inc.
Financial Services	AON	Aon plc
Financial Services	AXP	American Express Company
Financial Services	BAC	Bank of America Corporation
Financial Services	BEN	Franklin Resources, Inc.
Financial Services	BK	The Bank of New York Mellon Corporation
Financial Services	BLK	BlackRock, Inc.
Financial Services	C	Citigroup Inc.
Financial Services	CINF	Cincinnati Financial Corporation
Financial Services	CMA	Comerica Incorporated
Financial Services	CME	CME Group Inc.
Financial Services	FITB	Fifth Third Bancorp
Financial Services	GS	The Goldman Sachs Group, Inc.
Financial Services	HBAN	Huntington Bancshares Incorporated
Financial Services	HIG	The Hartford Financial Services Group, Inc.
Financial Services	ICE	Intercontinental Exchange, Inc.
Financial Services	IVZ	Invesco Ltd.
Financial Services	JPM	JPMorgan Chase & Co.
Financial Services	KEY	KeyCorp
Financial Services	L	Loews Corporation
Financial Services	LNC	Lincoln National Corporation
Financial Services	MCO	Moody's Corporation
Financial Services	MET	MetLife, Inc.
Financial Services	MMC	Marsh & McLennan Companies, Inc.
Financial Services	MS	Morgan Stanley
Financial Services	MTB	M&T Bank Corporation
Financial Services	NDAQ	Nasdaq, Inc.
Financial Services	NTRS	Northern Trust Corporation
Financial Services	PFG	Principal Financial Group, Inc.
Financial Services	PGR	The Progressive Corporation
Financial Services	PNC	The PNC Financial Services Group, Inc.
Financial Services	PRU	Prudential Financial, Inc.
Financial Services	RF	Regions Financial Corporation
Financial Services	RJF	Raymond James Financial, Inc.
Financial Services	SCHW	The Charles Schwab Corporation
Financial Services	SPGI	S&P Global Inc.
Financial Services	STT	State Street Corporation
Financial Services	TROW	T. Rowe Price Group, Inc.
Financial Services	TRV	The Travelers Companies, Inc.
Financial Services	UNM	Unum Group
Financial Services	USB	U.S. Bancorp
Financial Services	WFC	Wells Fargo & Company
Financial Services	ZION	Zions Bancorporation, National Association
Healthcare	A	Agilent Technologies, Inc.
Healthcare	ABC	AmerisourceBergen Corporation
Healthcare	ABT	Abbott Laboratories
Healthcare	ALGN	Align Technology, Inc.
Healthcare	AMGN	Amgen Inc.
Healthcare	BAX	Baxter International Inc.
Healthcare	BDX	Becton, Dickinson and Company
Healthcare	BIIB	Biogen Inc.
Healthcare	BMY	Bristol-Myers Squibb Company
Healthcare	BSX	Boston Scientific Corporation
Healthcare	CI	The Cigna Group
Healthcare	CNC	Centene Corporation
Healthcare	COO	The Cooper Companies, Inc.
Healthcare	CVS	CVS Health Corporation
Healthcare	DGX	Quest Diagnostics Incorporated
Healthcare	DVA	DaVita Inc.
Healthcare	EW	Edwards Lifesciences Corporation
Healthcare	GILD	Gilead Sciences, Inc.
Healthcare	HOLX	Hologic, Inc.
Healthcare	HSIC	Henry Schein, Inc.
Healthcare	HUM	Humana Inc.
Healthcare	IDXX	IDEXX Laboratories, Inc.
Healthcare	ILMN	Illumina, Inc.
Healthcare	INCY	Incyte Corporation
Healthcare	ISRG	Intuitive Surgical, Inc.
Healthcare	JNJ	Johnson & Johnson
Healthcare	LH	Laboratory Corporation of America Holdings
Healthcare	LLY	Eli Lilly and Company
Healthcare	MDT	Medtronic plc
Healthcare	MRK	Merck & Co., Inc.
Healthcare	MTD	Mettler-Toledo International Inc.
Healthcare	PFE	Pfizer Inc.
Healthcare	PRGO	Perrigo Company plc
Healthcare	REGN	Regeneron Pharmaceuticals, Inc.
Healthcare	RMD	ResMed Inc.
Healthcare	SYK	Stryker Corporation
Healthcare	TMO	Thermo Fisher Scientific Inc.
Healthcare	UHS	Universal Health Services, Inc.

Healthcare	UNH	UnitedHealth Group Incorporated
Healthcare	VRTX	Vertex Pharmaceuticals Incorporated
Healthcare	WAT	Waters Corporation
Healthcare	WBA	Walgreens Boots Alliance, Inc.
Healthcare	XRAY	DENTSPLY SIRONA Inc.
Healthcare	ZBH	Zimmer Biomet Holdings, Inc.
Industrials	AAL	American Airlines Group Inc.
Industrials	ADP	Automatic Data Processing, Inc.
Industrials	ALK	Alaska Air Group, Inc.
Industrials	AME	AMETEK, Inc.
Industrials	AOS	A. O. Smith Corporation
Industrials	BA	The Boeing Company
Industrials	CAT	Caterpillar Inc.
Industrials	CHRW	C.H. Robinson Worldwide, Inc.
Industrials	CMI	Cummins Inc.
Industrials	CSX	CSX Corporation
Industrials	CTAS	Cintas Corporation
Industrials	DE	Deere & Company
Industrials	DOV	Dover Corporation
Industrials	EFX	Equifax Inc.
Industrials	EMR	Emerson Electric Co.
Industrials	ETN	Eaton Corporation plc
Industrials	EXPD	Expeditors International of Washington, Inc.
Industrials	FAST	Fastenal Company
Industrials	FDX	FedEx Corporation
Industrials	FLS	Flowserve Corporation
Industrials	GD	General Dynamics Corporation
Industrials	GE	General Electric Company
Industrials	GPN	Global Payments Inc.
Industrials	GWW	W.W. Grainger, Inc.
Industrials	ITW	Illinois Tool Works Inc.
Industrials	JBHT	J.B. Hunt Transport Services, Inc.
Industrials	JCI	Johnson Controls International plc
Industrials	LMT	Lockheed Martin Corporation
Industrials	LUV	Southwest Airlines Co.
Industrials	MAS	Masco Corporation
Industrials	NOC	Northrop Grumman Corporation
Industrials	NSC	Norfolk Southern Corporation
Industrials	PAYX	Paychex, Inc.
Industrials	PCAR	PACCAR Inc
Industrials	PH	Parker-Hannifin Corporation
Industrials	PNR	Pentair plc
Industrials	PWR	Quanta Services, Inc.
Industrials	RHI	Robert Half Inc.
Industrials	ROK	Rockwell Automation, Inc.
Industrials	RSG	Republic Services, Inc.
Industrials	SWK	Stanley Black & Decker, Inc.
Industrials	TXT	Textron Inc.
Industrials	UNP	Union Pacific Corporation
Industrials	UPS	United Parcel Service, Inc.
Industrials	URI	United Rentals, Inc.
Industrials	WM	Waste Management, Inc.
Real Estate	O	Realty Income Corporation
Technology	AAPL	Apple Inc.
Technology	ACN	Accenture plc
Technology	ADBE	Adobe Inc.
Technology	ADI	Analog Devices, Inc.
Technology	ADSK	Autodesk, Inc.
Technology	AKAM	Akamai Technologies, Inc.
Technology	AMAT	Applied Materials, Inc.
Technology	AMD	Advanced Micro Devices, Inc.
Technology	ANSS	ANSYS, Inc.
Technology	APH	Amphenol Corporation
Technology	CDNS	Cadence Design Systems, Inc.
Technology	CRM	Salesforce, Inc.
Technology	CSCO	Cisco Systems, Inc.
Technology	CTSH	Cognizant Technology Solutions Corporation
Technology	DXC	DXC Technology Company
Technology	FFIV	F5, Inc.
Technology	FIS	Fidelity National Information Services, Inc.
Technology	GLW	Corning Incorporated
Technology	GRMN	Garmin Ltd.
Technology	HPQ	HP Inc.
Technology	IBM	International Business Machines Corporation
Technology	INTC	Intel Corporation
Technology	INTU	Intuit Inc.
Technology	IT	Gartner, Inc.
Technology	JNPR	Juniper Networks, Inc.
Technology	KLAC	KLA Corporation
Technology	LRCX	Lam Research Corporation
Technology	MCHP	Microchip Technology Incorporated
Technology	MSFT	Microsoft Corporation
Technology	MSI	Motorola Solutions, Inc.
Technology	MU	Micron Technology, Inc.
Technology	NTAP	NetApp, Inc.
Technology	NVDA	NVIDIA Corporation
Technology	ORCL	Oracle Corporation
Technology	QCOM	QUALCOMM Incorporated
Technology	ROP	Roper Technologies, Inc.
Technology	SNPS	Synopsys, Inc.
Technology	STX	Seagate Technology Holdings plc
Technology	SWKS	Skyworks Solutions, Inc.
Technology	TXN	Texas Instruments Incorporated
Technology	VRSN	VeriSign, Inc.
Technology	WDC	Western Digital Corporation
Utilities	AEE	Ameren Corporation
Utilities	AEP	American Electric Power Company, Inc.
Utilities	AES	The AES Corporation
Utilities	CMS	CMS Energy Corporation
Utilities	CNP	CenterPoint Energy, Inc.
Utilities	D	Dominion Energy, Inc.
Utilities	DTE	DTE Energy Company
Utilities	DUK	Duke Energy Corporation
Utilities	ED	Consolidated Edison, Inc.

Utilities	EIX	Edison International
Utilities	ES	Eversource Energy
Utilities	ETR	Entergy Corporation
Utilities	EXC	Exelon Corporation
Utilities	FE	FirstEnergy Corp.
Utilities	LNT	Alliant Energy Corporation
Utilities	NEE	NextEra Energy, Inc.
Utilities	NI	NiSource Inc.
Utilities	NRG	NRG Energy, Inc.
Utilities	PEG	Public Service Enterprise Group Incorporated
Utilities	PNW	Pinnacle West Capital Corporation
Utilities	SO	The Southern Company
Utilities	SRE	Sempra
Utilities	WEC	WEC Energy Group, Inc.
Utilities	XEL	Xcel Energy Inc.
